# Quantification of diastolic vortex shape deformation in left ventricular filling from 4D flow MRI

**DOI:** 10.1186/1532-429X-15-S1-P79

**Published:** 2013-01-30

**Authors:** Mohammed S ElBaz, Jos J Westenberg, Pieter J van den Boogaard, Boudewijn Lelieveldt, Rob J van der Geest

**Affiliations:** 1 Radiology, Leiden University Medical Center (LUMC), Leiden, the Netherlands

## Background

Vortical blood flow patterns (vortices) play a fundamental role in normal physiology of cardiac function in achieving the proper balance between blood flow and stress on surrounding structures and preventing blood stagnation. These patterns develop and evolve inside the cardiac atria and ventricles as a result of asymmetric chamber geometry. However, because of the complex geometry and flow patterns, their behavior and correlation to cardiac function is still under research. Moreover, most of the work in literature is based on qualitative analysis which has the drawbacks of subjectivity in interpretation and may impose observer bias towards the conclusions about these flow patterns. Accordingly, quantitative analysis of data is required for objective analysis of vortices.

The purpose of this study was to quantify the vortex shape deformation in left ventricular (LV) inflow patterns and to evaluate association with early and late filling flow velocity.

## Methods

Seven healthy volunteers (mean age: 40±15 years) underwent three-dimensional (3D), time resolved, three-directional velocity-encoded (VE) MR imaging at 1.5 T (Philips). VE MRI was performed in a 3D isotropic dataset of 4.2×4.2×4.2mm3 covering all 4 chambers of the heart. Retrospective gating with 30 phases reconstructed and velocity sensitivity of 150cm/s in all directions was used.

Vortical structures inside the LV were extracted from the velocity data using the Lambda2 method using a threshold of 3µ, with µ being the mean value of the voxels with λ2<0. Connected component analysis was applied to define and extract the vortical structures and to decrease the effect of noise. Detected components were considered a vortex when containing at least 800 voxels.The dimensions of the major large-scale vortex in the LV near the mitral annulus was further quantified at the moments of peak early (E) and late (A) filling rate. The shape of the LV vortex ring was quantified as the ratio between the short (L1) and long (L2) axes of the approximately doughnut shaped vortex ring.

Association between vortical shape and mean inflow velocity during early and late filling was evaluated.

## Results

**Figure 1:**
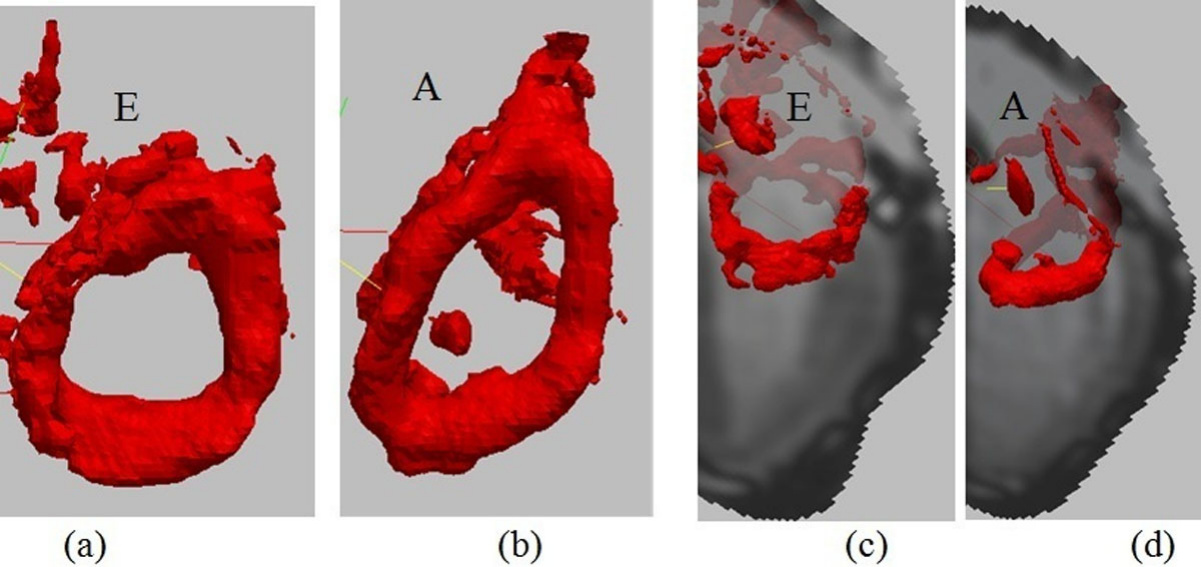
(a) Frame (16/30) represents the Early filling (E) peak frame and the LV vortex ring appears almost completely circular. (b) Frame (28/30) represents Late filling (A) peak in which vortex is elongated and no longer circular but of more elliptical shape. View from FH direction. (c,d) shows their positions in E and A frames in LA view and shows that they are at the same position.

**Figure 2:**
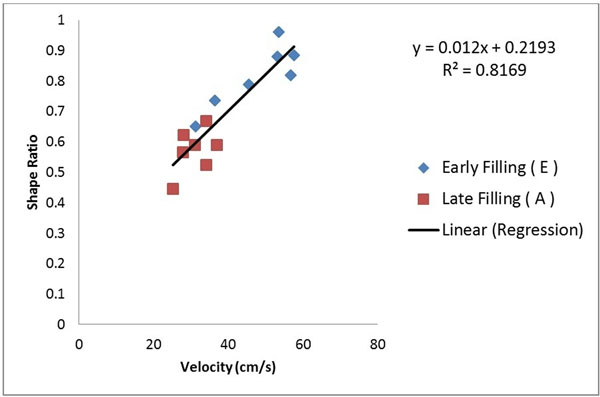
Correlation between shape ratio and early (blue) and late (red) filling velocity.

In each subject, an approximate LV vortex stretching (elongation) was observed at the moment of A filling rate relative to the E filling rate frame (Figure 1), in which the latter is more circular and the former is elongated in one direction relative to the other. The ratio L2/L1 at early peak filling rate was highly significantly different from the ratio L2/L1 at late peak filling rate (0.81±0.10vs 0.57±0.07, p<0.001). Furthermore, results show strong correlation(R2=0.81) between inflow velocity and vortical shape ratio (Figure 2).

## Conclusions

Intra-cardiac 4DFlow MRI consistently shows Early to Late filling LV vortex shape deformation in healthy volunteers. This deformation is quantified as the change in ratio between short and long axes of the LV vortex ring. This ring shape is associated with LV inflow velocity.

## Funding

Dutch Technology Foundation (STW): project number 11626

